# MOG Antibody Status Is Associated With Distinct Clinical Profiles and Treatment Responses in Chronic Relapsing Inflammatory Optic Neuropathy

**DOI:** 10.1212/NXI.0000000000200668

**Published:** 2026-10-22

**Authors:** Madalina Graure, Nathalie Nierobisch, Anthony De Vere-Tyndall, Thivya Pakeerathan, Ilya Ayzenberg, Jonathan A. Gernert, Joachim Havla, Marius Ringelstein, Orhan Aktas, Daria Tkachenko, Martin Werner Hümmert, Corinna Trebst, Nuria Cerdá-Fuertes, Athina Papadopoulou, Katrin Giglhuber, Rebecca Wicklein, Achim Berthele, Michael Weller, Veronika Kana, Patrick Roth, Marina Herwerth

**Affiliations:** 1 Department of Neurology, University Hospital Zurich, Switzerland;; 2 Neuroscience Center Zurich, University of Zurich and ETH Zurich, Switzerland;; 3 Department of Diagnostic and Interventional Neuroradiology, University Hospital Zurich, Switzerland;; 4 Department of Neurology, St. Josef Hospital, Ruhr University Bochum, Germany;; 5 Institute of Clinical Neuroimmunology, LMU University Hospital, Ludwig-Maximilians-University Munich, Germany;; 6 Department of Neurology, Medical Faculty and University Hospital, Heinrich Heine University Düsseldorf, Germany;; 7 Department of Neurology, Centre for Neurology and Neuropsychiatry, LVR-Klinikum, Heinrich Heine University Düsseldorf, Germany;; 8 Department of Neurology, Hannover Medical School, Germany;; 9 PRACTIS Clinician Scientist Program, Dean's Office for Academic Career Development, Hannover Medical School, Germany;; 10 Department of Neurology, Klinikum Agnes Karl Laatzen/ Klinikum Region Hannover, Germany;; 11 Department of Neurology, University Hospital Basel, Switzerland;; 12 Department of Clinical Research, University of Basel, Switzerland;; 13 Translational Imaging in Neurology (ThINK) Basel, Department of Medicine and Biomedical Engineering, University Hospital Basel and University of Basel, Switzerland;; 14 Department of Neurology, School of Medicine and Health, Technical University Munich, Germany; and; 15 Institute of Pharmacology and Toxicology, University of Zurich, Switzerland.

## Abstract

**Background and Objectives:**

Chronic relapsing inflammatory optic neuropathy (CRION) is a steroid-dependent form of optic neuritis with incompletely understood pathophysiology. The identification of myelin oligodendrocyte glycoprotein antibodies (MOG-IgG) in a substantial patient subset has challenged the diagnostic and therapeutic management. The aim of this study was to investigate clinical profiles and treatment outcomes of patients with CRION, comparing MOG-IgG-positive (MOG+) and seronegative (MOG−) subgroups.

**Methods:**

Patients from 6 European tertiary centers fulfilling diagnostic criteria for CRION were included. All underwent cell-based autoantibody testing. Clinical outcomes (visual acuity and annualized relapse rate), laboratory and imaging findings (MRI, OCT), and treatment responses were retrospectively analyzed.

**Results:**

Sixty patients were included (median age 33 years; 70% female); 27 (45%) were MOG+. MOG+ CRION was associated with later onset, higher annualized relapse rate (ARR) before treatment (median [IQR] 2 [1–3] vs 1 [1–2], *p =* 0.023), and a trend toward shorter interrelapse intervals. Additional distinguishing features included higher frequencies of antinuclear antibody positivity, elevated CSF interleukin-6, and extensive enhancement of the optic nerve on MRI. Relapse burden correlated with visual acuity decline and retinal thinning. In MOG+ patients, monoclonal antibody therapy reduced the ARR (n = 21; 2 [1–3] vs 0 [0–2], *p* = 0.024), primarily driven by tocilizumab (n = 11; 2 [1–3] vs 0 [0–1], *p =* 0.023). In MOG− patients, rituximab and azathioprine showed a trend toward ARR reduction.

**Discussion:**

CRION represents a heterogeneous syndrome encompassing distinct subgroups. Although MOG+ patients demonstrate higher disease activity, tocilizumab might represent a therapeutic option, though further validation is needed. Serologic testing is critical for treatment stratification and preventing relapses.

## Introduction

Chronic relapsing inflammatory optic neuropathy (CRION), a rare, steroid-dependent form of optic neuritis (ON), remains poorly understood.^1-3^ A key challenge is that even after excluding alternative diagnoses, CRION is identified only when acute therapy is withdrawn leading to a pathognomonic relapse, rather than at presentation. Steroid tapering in the treatment of ON is not routinely practiced,^4^ potentially leaving high-risk patients vulnerable to preventable relapses. This diagnostic delay poses a significant risk of irreversible visual acuity (VA) worsening and highlights the urgent need for a better characterization and management of this rare disease.

Advances in antibody testing, particularly for aquaporin-4 (AQP4-IgG) and myelin oligodendrocyte glycoprotein (MOG-IgG), have raised debate over whether CRION is a distinct entity because previous studies found MOG-IgG positivity (MOG+) in 25–90% and AQP4-IgG seropositivity in 5–25% of patients fulfilling the diagnostic criteria for CRION.^5-7^

Recommendations for maintenance steroid-sparing therapy remain a considerable challenge in clinical practice and are based on case reports or monocentric studies with no standardized treatment protocols.^8,9^ A spectrum of immunosuppressive agents, monoclonal antibodies, and IV immunoglobulins (IVIG) have been proposed as therapeutic options.^10-13^ However, longitudinal clinical characterization, therapy response, and outcome of CRION subtypes remain underexplored.

The aim of this retrospective, multicenter study was to compare clinical profiles and treatment responses in patients with the MOG+ vs MOG− CRION phenotype. Outcomes included visual acuity, laboratory and imaging features, and annualized relapse rates across treatment regimens stratified by MOG− Ab status.

## Methods

### Study Design and Patient Selection

This retrospective, longitudinal, multicenter study included patients older than 16 years from 6 tertiary referral centers in Switzerland (n = 1) and Germany (n = 5) who met the diagnostic criteria for CRION,^2^ which require the fulfillment of the following criteria: (1) history of ON and at least 1 relapse, (2) clinical objective evidence for loss of visual function, (3) NMO-IgG seronegativity, (4) contrast enhancement on the MRI of the acutely inflamed optic nerves, and (5) response to immunosuppressive treatment and relapse on withdrawal or dose reduction of immunosuppressive treatment. Medical records and Neuromyelitis Optica Study Group database entries from January 1, 2010, to June 30, 2024, were screened for the keywords “CRION,” “chronic relapsing inflammatory optic neuropathy,” or “relapsing optic neuritis,” yielding 5,507 screened individuals, of whom 102 met initial screening criteria (eFigure 1).

A relapse was defined as an objective worsening in VA persisting for ≥24 hours in the absence of an alternative etiology (e.g., infection) and supported by clinical and/or radiologic findings. Relapses within 2 months were classified as early relapses. Given the lack of consensus on the minimum time interval required to distinguish separate relapse events in CRION, no minimum interrelapse interval was defined a priori. Completeness of records required documentation of VA assessment, at least 1 brain MRI, MOG-IgG and AQP4-IgG testing, screening for rheumatologic and infectious diseases, and CSF analysis.

Exclusion criteria included demyelinating lesions outside the optic nerves, diagnoses of MS, AQP4-IgG-positive or seronegative neuromyelitis optica spectrum disorder, rheumatologic (e.g., systemic lupus erythematosus, Sjögren syndrome, sarcoidosis, Behçet disease, and vasculitis) or infectious disease at the initial presentation or during any follow-up, as well as incomplete diagnostic workup. Following detailed review, 42 individuals were excluded (eFigure1). For comparison of optical coherence tomography (OCT) outcomes, 30 age-matched and sex-matched healthy controls without neurologic or ophthalmologic disease were recruited from a Swiss tertiary referral center.

Outcomes included visual acuity, laboratory and imaging features, and annualized relapse rates across treatment regimens stratified by MOG-Ab status.

### Clinical and Laboratory Data

Except for 1 individual, all patients underwent at least 2 independent cell-based assays for MOG-IgG; all patients were screened for AQP4-IgG. Baseline testing was conducted before the administration of acute relapse therapies, such as IVIG or corticosteroids. The neutrophil-to-lymphocyte ratio (NLR) was determined within 28 days of ON onset and before steroid treatment. If unavailable for the first attack, the earliest NLR data from a subsequent relapse were collected. CSF analysis included cell count and differentiation, albumin quotient, oligoclonal bands, lactate, and CSF/serum glucose ratio. CSF MOG-IgG screening was not routinely performed. IL-6 measurements were performed using the Human IL-6 High Sensitivity Magnetic Luminex® Performance Assay, R&D Systems.

### Magnetic Resonance Imaging

The first centrally available 3 Tesla MRI scans were independently reviewed by 2 neuroradiologists blinded for the MOG antibody status. Protocols included T1-weighted (± gadolinium), T2-weighted, and FLAIR brain sequences, as well as gadolinium-enhanced and fat-suppressed T2-weighted orbital sequences. Optic nerve localization and extent, perineural involvement, and extraorbital parenchymal lesions were assessed.

### Visual Acuity

High-contrast visual acuity VA was measured using standardized Early Treatment Diabetic Retinopathy Study or Snellen charts. To assess long-term disease burden, only measurements ≥8 weeks after the most recent ON episode were included, with most of them performed >12 weeks.

### Optical Coherence Tomography

Retinal imaging was performed with the SPECTRALIS Spectral Domain-OCT (Heidelberg Engineering) with automatic real-time tracking function. Peripapillary retinal nerve fiber layer (pRNFL) was assessed with a 3.4 mm ring scan, 12°, centered on the optic nerve head. The combined thickness of the ganglion cell layer and the inner plexiform layer (GCIPL) was evaluated from macular scans 6 mm diameter annulus centered on the fovea (61 B-scans, scanning angle 30° × 25°). Volumes were calculated by the software's segmentation algorithm and converted into thickness through division by the scanned surface area. Scan quality was verified with OSCAR-IB criteria.^14^ All images were analyzed using the HEYEX software suite (versions 2.5.5, 2.6.4, or 2.6.9; Heidelberg Engineering), which uses consistent automated segmentation algorithms across versions. Cross-sectional analyses used the most recent OCT ≥8 weeks after ON onset.

### Treatment Effectiveness

Therapies were classified as acute (corticosteroids pulse and plasmapheresis), postacute management (corticosteroid-tapering), or long-term steroid-sparing. The latter included azathioprine, rituximab, tocilizumab, mycophenolic acid, IVIG, and other agents. For statistical analysis, long-term regimens with ≥5 documented cases were grouped into monoclonal antibodies (rituximab and tocilizumab) and immunosuppressants (azathioprine and mycophenolic acid).

### Statistical Analysis

Statistical analyses were performed using R 4.3.2 and GraphPad Prism 10.3.1. Nonparametric tests were applied because of small subgroup sizes and nonnormal distributions. Differences were considered statistically significant at *p* < 0.05. Unless stated otherwise, nominal *p* values were applied. Results are presented as medians with interquartile ranges [IQR], or as absolute numbers and percentages of total. The Fisher exact test was used for categorical variables, Mann-Whitney *U* for independent samples, and Wilcoxon signed-rank for paired data. For multiple comparison, we applied the Kruskal-Wallis test followed post hoc by the Dunn test.

Correlations between continuous variables (e.g., VA and number of ON episodes) were assessed by linear regression. The association of multiple predictors with an outcome was assessed using a linear mixed-effects model.

VA outcome analyses were limited to affected eyes (eyes with clinical and instrumental diagnostic evidence of at least 1 ON episode). Asymptomatic structural findings or noninflammatory abnormalities detected on OCT or MRI were not classified “affected.” Individual trajectories under steroid-sparing therapy were visualized through swimmer plots; patients with unknown relapse intervals were excluded.

The annualized relapse rate (ARR) for each patient was calculated as the number of relapses per year. To minimize overestimation of therapy efficacy, patients with less than 12 months of follow-up after therapy initiation were excluded. To account for variability in follow-up duration, ARR was compared over fixed 12-month intervals immediately before and after treatment initiation. Comparisons were performed according to mechanism of action (monoclonal antibodies and immunosuppressants) and for individual regimens with at least 5 treated cases.

Missing data were handled using a complete-case approach, with no imputation performed. Analyses were restricted to the relevant subpopulations for which data were available.

### Ethical Considerations

Ethical approval was obtained from the Ethics Board of the Canton of Zurich (KEK-ZH-Nr. 2024-00239). All participants provided written informed consent.

### Data Availability

The data that support the findings of this study are available from the corresponding author, on reasonable request.

## Results

### Study Participants

Sixty patients who met the diagnostic criteria for CRION were included. Of these, 27 individuals (45%) tested positive for MOG-IgG through cell-based assays, therefore fulfilling diagnostic criteria for MOG antibody-associated disease (MOGAD).^15^ Among the seronegative patients (n *=* 33/60; 55%), no autoantibodies (MOG-IgG, AQP4-IgG) were detected despite repeated testing. Antibody status was determined using fixed CBA (83.3% of individuals) or live CBA (55%), with some patients undergoing both testing modalities. Both MOG+ and MOG− CRION patients (Table 1) were predominantly female, exhibited a median of 3 ON episodes, and presented with similar low percentage of eyes classified as unaffected by ON. MOG+ CRION individuals were older at onset (median [IQR] 38 years [32–50], vs 28 years [24.5–39.5] for MOG−, *p =* 0.015; eFigure 2A). Although the median ARR over the total follow-up period was similar between both subgroups (MOG+: 0.9 [0.5–1.2], MOG−: 0.8 [0.5–1.3]), the median [IQR] ARR in the 12 months before therapy initiation in the MOG+ CRION group was significantly higher compared with the MOG− CRION subgroup (2 [1–3] vs 1 [1–2], *p =* 0.023; eFigure 2B). Early recurrence of ON (inter-ON interval <2 months) was observed in 39.3% of the total cohort with a trend toward higher occurrence in the MOG+ vs the MOG− subgroup (14/26 [53.9] vs 8/30 [26.7]; *p =* 0.055; eFigure 2C). The median [IQR] duration between relapses and across all patients was 4.9 [2–12.3] months, with a minimum interval of 2 weeks in one case. Prodromal eye pain and color desaturation were present in comparable percentages in both groups. In the MOG+ vs MOG− subgroups, prodromal eye pain was present in n = 25/27 (92.6%) vs n = 27/31 (87.1%) of individuals, *p* = 0.675. Color desaturation was present in MOG+ vs MOG−: n = 14/16 (87.5) vs n = 19/30 (63.3%), *p* = 0.101. By contrast, papilledema was significantly more frequent in MOG+ CRION cases: n = 11/17 (64.7%) vs n = 5/24 (20.8%), *p* = 0.009, Table 1.

**Table 1:** Demographic, Clinical, Laboratory, and MRI Findings in MOG+ vs MOG− CRION

N (%)	Total CRION 60 (100)	MOG+ 27 (45)	MOG− 33 (55)	*p* Value
Clinical parameters				
Sex F, n/N (%)	42 (70)	17 (63)	25 (75.8)	0.397
Age at onset, yr, median [IQR]	33 [25.3–44.5]	38 [32–50]	28 [24.5–39.5]	0.015
Total FU, mo, median [IQR]	50.5 [20.5–97.5]	56 [13–109]	45 [25–90.5]	0.760
ON episodes, median [IQR]	3 [2–5]	3 [2–6.3]	3 [2–4]	0.264
Noneyes, n/N (%)	13/120 (10.8)	5/54 (9.3)	8/66 (12.1)	0.770
Prodromal eye pain n/N (%)	52/58 (89.7)	25/27 (92.6)	27/31 (87.1)	0.675
Color desaturation (%)	33/46 (71.7)	14/16 (87.5)	19/30 (63.3)	0.101
Papilledema (%)	16/41 (39)	11/17 (64.7)	5/24 (20.8)	0.009
ARR/total FU, median [IQR]	0.8 [0.5–1.3]	0.9 [0.5–1.2]	0.8 [0.5–1.3]	0.916
ARR 12 mo before therapy, median [IQR] (n = 51)	2 [1–3]	2 [1–3]	1 [1–2]	0.023
Patients with early recurrent ON^a^, n/N (%)	22/56 (39.3)	14/26 (53.9)	8/30 (26.7)	0.055
VA outcome affected eyes, decimal, median [IQR] (*n =* 71)	0.8 [0.4–1]	0.9 [0.4–1]	0.8 [0.3–0.9]	0.358
Laboratory findings at baseline				
Serum				
Positive ANA, n/N (%)	15/53 (28.3)	10/21 (47.6)	5/32 (15.6)	0.015
NLR, median [IQR] (*n =* 20)	2.4 [2.1–3.1]	2.4 [2.2–3.5]	2.1 [1.9–2.7]	0.289
Cerebrospinal fluid				
Elevated WBCC, n/N (%)	9/50 (18)	5/22 (22.7)	4/28 (14.3)	0.482
WBCC/μL, median [IQR] (*n =* 50)	2 [1–4]	2 [1.8–4.5]	2 (1–4)	0.178
Neutrophil detection, n/N (%)	3/24 (12.5)	3/10 (30)	0/14 (0)	0.059
Elevated QAlb, n/N (%)	12/51 (23.5)	7/22 (31.8)	5/29 (17.2)	0.320
Positive OCB, n/N (%)	9/58 (15.5)	3/25 (12)	6/33 (18.2)	0.718
Elevated lactate, n/N (%)	3/35 (8.6)	2/16 (12.5)	1/19 (5.3)	0.582
Low glucose index, n/N (%)	1/33 (3)	0/14 (0)	1/19 (5.3)	>0.999
IL-6 pg/mL, median [IQR] (*n =* 14)	7 [1.6–67.5]	50.9 [2.3–89]	1.7 [0.7–7]	0.044
MRI findings at baseline				
Dedicated orbital scans N (%)	22/23 (95.7)	15/16 (93.8)	7/7 (100)	>0.999
Contrast enhancement of optic nerve, n/N (%)	20/23 (87)	14/16 (87.5)	6/7 (85.7)	>0.999
T2 hyperintensity of optic nerve, n/N (%)	21/23 (91.3)	15/16 (93.8)	6/7 (85.7)	0.526
ON localization				
Orbital, n/N (%)	22/23 (95.7)	16/16 (100)	6/7 (85.7)	0.304
Canalicular, n/N (%)	23/23 (100)	16/16 (100)	7/7 (100)	>0.999
Chiasmal, n/N (%)	6/23 (26.1)	4/16 (25)	2/7 (28.6)	>0.999
ON characteristics				
Extensive (>1/2), n/N (%)	20/23 (87)	16/16 (100)	4/7 (57.1)	0.020
Bilateral, n/N (%)	5/23 (21.7)	4/16 (25)	1/7 (14.3)	>0.999
Perineural enhancement, n/N (%)	10/23 (43.5)	9/16 (56.3)	1/7 (14.3)	0.089

Abbreviations: ANA = antinuclear antibodies; ARR = annualized relapse rate; IQR = interquartile range; FU = follow-up; IL-6 = interleukin 6; mo = months; MOG+ = CRION patients who tested positive for MOG-IgG; MOG− = CRION patients who tested negative for MOG-IgG; n/N = number of cases (n) out of the total number assessed (N); NLR = neutrophil-to-lymphocyte ratio; noneyes = eyes without a history of ON; OCB = oligoclonal bands; ON = optic neuritis episode; QAlb = cerebrospinal fluid/serum albumin quotient; T2 = T2-weighted fat-suppressed MRI sequence; VA = visual acuity; WBCC = white blood cell count; yr = years.

a Patients with early recurrent ON = patients in which at least 1 interval between relapses was <2 mo.

### Laboratory Findings

A significantly higher proportion of MOG+ CRION individuals showed elevated antinuclear antibody (ANA) titers (10/21; 47.6%) compared with the MOG− CRION subgroup (5/32; 15.6%; *p* = 0.015, eFigure 2F). ANA positivity did not correlate with the number of relapses (*p =* 0.946). Measurement of the NLR within 28 days of relapse onset trended toward higher values in the MOG+ CRION compared with the MOG− CRION subgroup (n *=* 20; median [IQR] 2.4 [2.2–3.5] for MOG+ vs 2.1 [1.9–2.7] for MOG−; *p* = 0.289).

CSF analysis revealed pleocytosis in 9 of 50 patients (18%) of the total CRION cohort, with comparable frequencies and a tendency toward higher cell counts in the MOG+ subgroup (eFigure 2D). Positive oligoclonal bands were detected in a minority of patients with similar distribution across subgroups. The MOG+ subgroup exhibited significantly higher CSF IL-6 levels than MOG− CRION patients (n *=* 14; median [IQR] for MOG+ 50.9 [2.3–89] vs 1.7 [0.7–7] in MOG−; *p =* 0.044; eFigure 2E). Neutrophils were observed in the CSF of MOG+ individuals but were absent in the MOG− subgroup (3/10 [30%] for MOG+ vs 0/14 [0%] for MOG−; *p =* 0.059).

### Radiologic Findings

MRI data were available for 23 patients. Dedicated orbital MRI scans (T1-weighted, gadolinium-enhanced fat-suppressed T1-weighted, fat-suppressed T2-weighted, and fluid-attenuated inversion recovery [FLAIR] sequences) were available for most patients (95.7%). Scans were generally acquired during an acute or subacute ON episode to assess orbital involvement (median [IQR] of 0 [0–1] months from symptom onset), in accordance with standard clinical practice.

Extensive optic nerve involvement, defined as lesions affecting more than half of the nerve length, was significantly more common in MOG+ CRION individuals compared with the MOG− subgroup (16/16 [100%] vs 4/7 [57.1%]; *p =* 0.020, Table 1; eFigure 2H). MOG+ CRION cases exhibited a higher tendency for perineural contrast enhancement compared with MOG− cases, although the difference did not reach statistical significance (9/16 [56.3%] vs 1/7 [14.3%]; *p =* 0.089; eFigure 2I).

There were no differences in localization patterns between the MOG+ and MOG− CRION subgroups (Table 1). All patients (100%) exhibited involvement of the canalicular segment, while the orbital segment was affected in most cases (95.7%). Affection of the optic chiasm was observed in 6 patients (26.1%).

### Visual Outcome

Analysis of VA of affected eyes (n *=* 71) in individuals with CRION revealed that a higher number of ON episodes correlated with poorer VA outcomes (B = −0.036, 95% CI −0.065 to −0.006, *p =* 0.018), while worsening was accentuated in the MOG+ subgroup (Figure 1). Analysis restricted to eyes with VA assessments performed ≥3 months after the last ON episode yielded comparable results, with a modest reduction in statistical power (n = 63 eyes; B = −0.032, 95% CI −0.063 to −0.001, *p* = 0.044).

**Figure 1:**
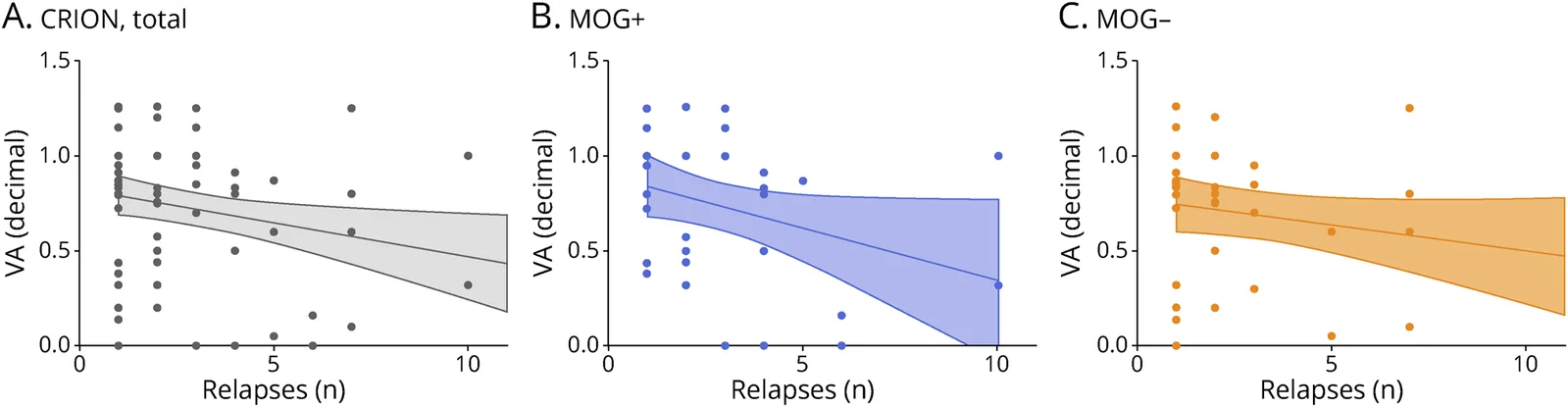
Visual Acuity Outcome of Affected Eyes in Patients With CRION (A–C) Scatterplots with linear regression lines and 95% CI (light-shaded bands) illustrating the relationship between the visual acuity outcome (decimal) and the number of relapses (A) in the total cohort, (B) in the MOG+ subgroup, and (C) in the MOG− subgroup. Simple linear regression, *p =* 0.018 (A), *p =* 0.055 (B), *p =* 0.126 (C); each dot represents an affected eye. MOG+ = CRION patients who tested positive for MOG-IgG; MOG− = CRION patients who tested negative for MOG-IgG; n *=* number; VA = visual acuity.

Early relapse recurrence or higher IL-6 levels were not predictive of poorer VA outcome (*p =* 0.679 and *p =* 0.090, respectively), although the latter showed a potential trend.

### Optical Coherence Tomography Findings

OCT cross-sectional data were available in 42 of 60 (70%) of included individuals with CRION. In the MOG+ subgroup, progressive thinning of both pRNFL and GCIPL was observed with increasing number of ON episodes (pRNFL in MOG+: healthy controls vs 1-ON vs ≥2 ON, n *=* 41 eyes, median [IQR] pRNFL 100 [98–106] vs n *=* 14, median [IQR] 76.5 [60.8–85.5] vs n *=* 23, median [IQR] 55 [39–72], *p* < 0.001 for both comparisons with healthy controls; GCIPL in MOG+: n *=* 40 eyes, median [IQR] GCIPL 86.3 [83.5–91.9] vs n *=* 14, median [IQR] 75 [54.5–83.5] vs n *=* 23, median [IQR] 62.2 [46–77.4], *p* = 0.001 for both comparisons, pRNFL thinning Figure 2, GCIPL thinning eFigure 3).

**Figure 2:**
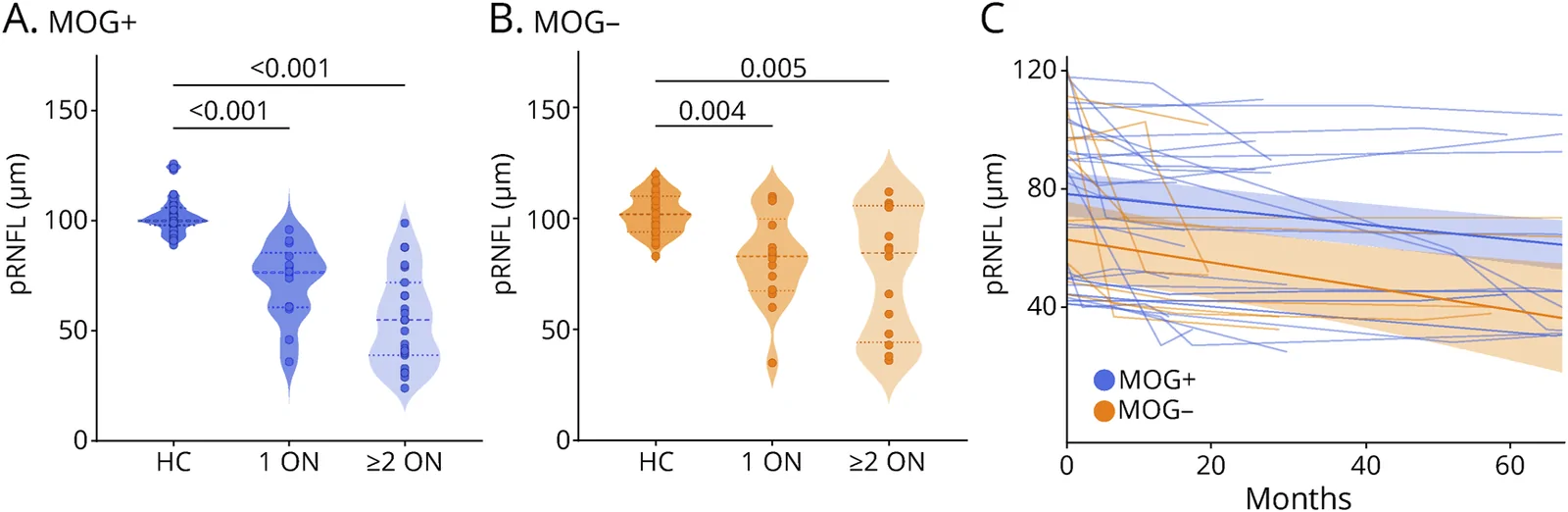
Temporal Changes in OCT-Based pRNFL Thickness Across CRION Subgroups (A and B) Violin plots illustrating the median, IQR, and range of pRNFL thickness in µm in healthy controls compared with MOG+ (A) and MOG− (B) patients with 1 ON and ≥2 ON, respectively (Kruskal-Wallis test, followed post hoc by the Dunn test). Each dot represents a single eye. (C) Longitudinal pRNFL thickness trajectories from MOG+ patients (blue) and MOG− CRION patients (orange) predicted by the linear mixed-effects model (thick lines = median; light-shaded bands = 95% CI). Thin lines show individual eye-based trajectories, plotted over the maximum follow-up duration. HC = healthy controls; MOG+ = CRION patients who tested positive for MOG-IgG; MOG− = CRION patients who tested negative for MOG-IgG; pRNFL = peripapillary retinal nerve fiber layer.

In the MOG− subgroup, both pRNFL and GCIPL values were also reduced compared with healthy controls (pRNFL: healthy controls vs 1-ON vs ≥2 ON, n *=* 35 eyes, median [IQR] pRNFL 102 [94–110] vs n *=* 14, median [IQR] 83 [67.5–99.8] vs n *=* 16, median [IQR] 84.5 [44.3–105.8], *p* = 0.004 and 0.005, respectively; GCIPL: n *=* 33 eyes, median [IQR] GCIPL 88.4 [82.4–93] vs n *=* 8, median [IQR] 50.9 [28.3–69.3] vs n *=* 7, median [IQR] 67.9 [48.1–84.9], *p* < 0.001 and *p* = 0.010, respectively, pRNFL thinning Figure 2, GCIPL thinning eFigure 3).

However, unlike in the MOG+ subgroup, progressive thinning with increasing ON episodes was not consistently observed in MOG− patients. This finding in the cross-sectional analysis is likely attributable to the limited sample size and greater variability within the MOG− subgroup. Therefore, longitudinal OCT data were analyzed to further assess retinal structural changes over time. In patients with longitudinal data (23/60, n *=* 45 eyes), both pRNFL and GCIPL layers showed a progressive thinning in the MOG+ and MOG− subgroups (Figure 2C, eFigure 3C). The high ARR, with a median [IQR] of 0.8 [0.5–1.3] ON attacks per year during the follow-up period, precluded a longitudinal analysis independent of ON episodes. Thicknesses declined for both pRNFL (n *=* 27 eyes, estimate, B = −0.252, standard error, SE = 0.054, *p* < 0.001) and GCIPL (n *=* 19 eyes, B = −0.178, SE = 0.050, *p* < 0.001) in MOG+ CRION patients over time, as did pRNFL (n *=* 10 eyes, B = −0.389, SE = 0.135, *p* = 0.005) and GCIPL (n *=* 5 eyes, B = −0.439, SE = 0.180, *p =* 0.018) in MOG− CRION patients. There was no significant difference in thinning rates between the groups (pRNFL: *p =* 0.347, GCIPL: *p =* 0.167).

To account for prolonged thinning of retinal layers after ON, a sensitivity analysis including only OCT examinations performed ≥6 months after the last ON attack was conducted, demonstrating consistent ongoing thinning of both pRNFL and GCIPL (eFigure 4).

### Treatment Choice and Effectiveness

All patients received corticosteroids during acute ON episodes and subsequently demonstrated improvement in VA, as required by the inclusion criteria (Table 2). The median number of 3 corticosteroid courses per patient, and the proportion undergoing plasmapheresis, e.g., in severe cases or due to recurrent VA decline post–steroid-tapering, were comparable between subgroups (8/27 [29.6%] for MOG+ vs 11/33 [33.3%] for MOG−; Table 2).

**Table 2:** Acute and Maintenance Therapy of Patients With CRION

Therapy	Total CRION	MOG+	MOG−	*p* Value
No. of CS courses per patient, median [IQR]	3 [2–4]	3 [2–6]	3 [2–4]	0.369
Plasmapheresis, n/N (%)	19/60 (31.7)	8/27 (29.6)	11/33 (33.3)	0.788
No steroid sparing therapy, n/N (%)	19/60 (31.7)	7/27 (25.9)	12/33 (36.4)	0.419
Steroid-sparing therapy				
Azathioprine, n/N (%)	19/41 (46.3)	9/20 (45)	10/21 (47.6)	>0.999
Rituximab, n/N (%)	16/41 (39)	10/20 (50)	6/21 (28.6)	0.118
Tocilizumab, n/N (%)	13/41 (31.7)	12/20 (60)	1/21 (4.8)	<0.001
Mycophenolic acid, n/N (%)	5/41 (12.2)	3/20 (15)	2/21 (9.5)	0.663
IVIG, n/N (%)	4/41 (9.8)	4/20 (20)	0/21 (0)	0.042
Dimethyl fumarate, n/N (%)	3/41 (7.3)	0/20 (0)	3/21 (14.3)	0.232
Glatiramer acetate, n/N (%)	2/41 (4.9)	0/20 (0)	2/21 (9.5)	0.488
Interferon, n/N (%)	2/41 (4.9)	0/20 (0)	2/21 (9.5)	0.488
Methotrexate, n/N (%)	1/41 (2.4)	1/20 (5)	0/21 (0)	0.488
Teriflunomid, n/N (%)	1/41 (2.4)	0/20 (0)	1/21 (4.8)	>0.999
Cyclophosphamide, n/N (%)	1/41 (2.4)	1/20 (5)	0/21 (0)	0.488
Inebilizumab, n/N (%)	1/41 (2.4)	0/20 (0)	1/21 (4.8)	>0.999
Fingolimod, n/N (%)	1/41 (2.4)	0/20 (0)	1/21 (4.8)	>0.999
Natalizumab, n/N (%)	1/41 (2.4)	1/20 (5)	0/21 (0)	0.488
Combination therapy, n/N (%)	2/41 (4.9)	2/20 (10)	0/21 (0)	0.232

Abbreviations: CS = corticosteroids; combination therapy = concomitant treatment with 2 agents; IVIG = IV immunoglobulin; MOG+ = CRION patients who tested positive for MOG-IgG; MOG− = CRION patients who tested negative for MOG-IgG; n/N = number of cases (n) out of the total number assessed (N).

Nineteen of 60 patients with CRION (31.7%) did not receive any long-term steroid-sparing therapy (7/27 [25.9] for MOG+ vs 12/33 [36.4] for MOG−; *p =* 0.419). Adjustment of treatment strategies was required more often in MOG+ CRION cases, with individuals receiving a median [IQR] of 1.5 [1–2.3] therapies vs 1 [1-1] in MOG− (*p =* 0.041; Figure 3). Azathioprine (19/41 [46.3%]) and rituximab (16/41; [39%]) were the most commonly used agents across both CRION subgroups (Table 2). Tocilizumab was preferentially initiated in MOG+ CRION patients, while IVIG (1–2 g/kg of body weight, every 4 weeks) was administered exclusively to individuals in the MOG+ subgroup. Mycophenolic acid was prescribed in (5/41 [12.2%]) of the overall cohort, with similar distribution between subgroups. Dimethyl fumarate was used exclusively in (3/21 [14.3%]) of MOG− CRION patients. Other therapies were used infrequently across the cohort (Table 2).

**Figure 3:**
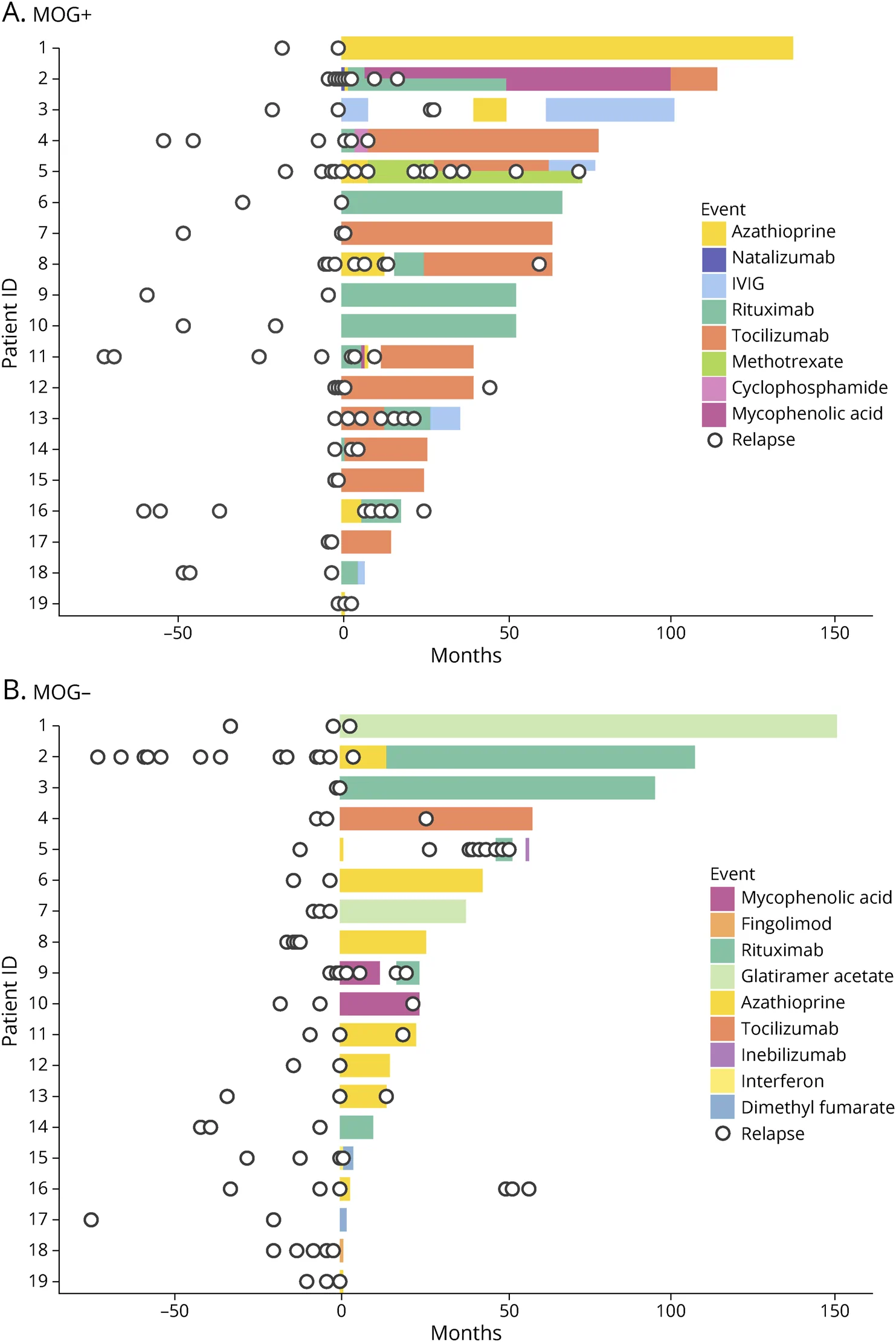
Longitudinal Disease Courses and Individual Maintenance Therapies Across CRION Subgroups (A and B) Swimmer plot illustrating individual disease trajectories in MOG+ and MOG− individuals. Attacks are indicated as circles. IVIG = IV immunoglobulin; MOG+ = CRION patients who tested positive for MOG-IgG; MOG− = CRION patients who tested negative for MOG-IgG.

Concomitant treatment with 2 steroid-sparing agents was administered exclusively within the MOG+ CRION subgroup (2/20; [10%]). One patient received a combination of rituximab and mycophenolic acid. Another patient was initially treated with methotrexate with concomitant tocilizumab, which was later discontinued because of relapse; subsequent management included a regimen of methotrexate, IVIG, and low-dose corticosteroids. Individual longitudinal disease courses and maintenance immunotherapies for both MOG+ and MOG− CRION subgroups are illustrated in Figure 3.

In 28 of 51 cases (54.9%), there was at least 1 documented relapse within 12 months after initiation of a new steroid-sparing therapy. ARR for the entire cohort declined significantly following initiation of maintenance therapy, regardless of treatment type (median [IQR] 2 [1–3] pretreatment vs 1 [0–2] posttreatment; *p =* 0.003). Analysis of treatment strategies yielded a significant association between initiation of monoclonal antibodies (encompassing rituximab and tocilizumab) in MOG+ patients and ARR reduction (n *=* 21; median [IQR] before vs after: 2 [1–3] vs 0 [0–2], *p =* 0.024). In patients receiving immunosuppressive therapy (n *=* 10), the ARR remained unchanged (median [IQR] before 2 [0–3] vs after 2 [0–3]; *p =* >0.999; Figure 4A). All 4 MOG+ individuals treated with IVIG remained relapse-free during follow-up; however, this subgroup did not meet the prespecified criteria for formal analysis (≥5 patients and ≥12 months of follow-up before and after initiation).

**Figure 4:**
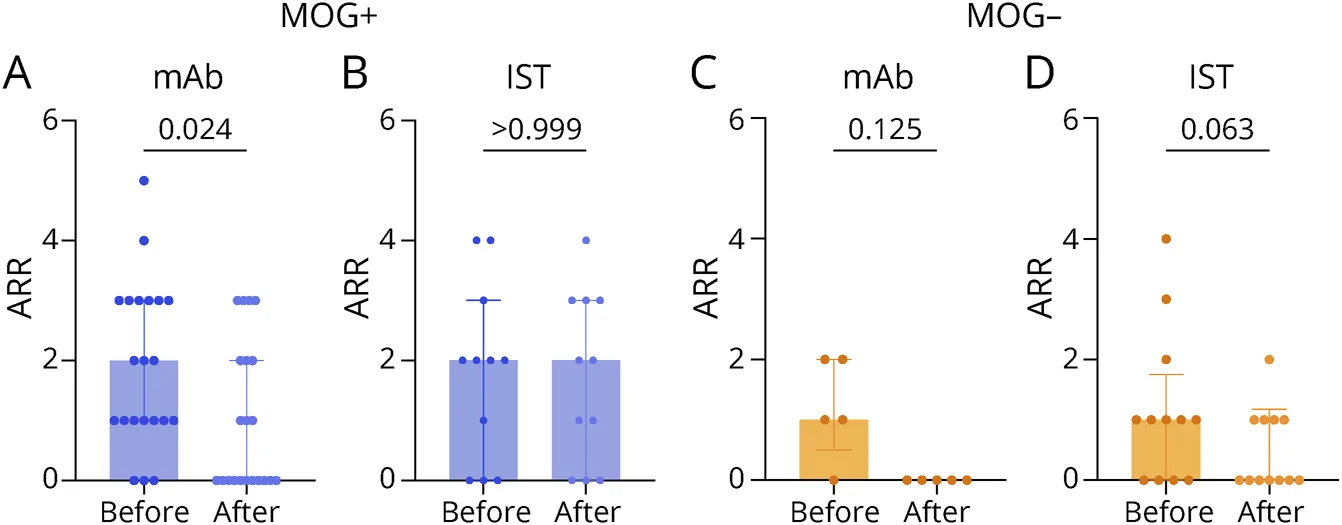
Annualized Relapse Rate Before and After Maintenance Therapy Initiation (A-D) Box plots illustrating median and IQR of the ARR in the MOG+ subgroup (A-B) and MOG− subgroup (C-D), comparing the 12 months before and after initiation of the 4 most used treatment strategies: immunosuppressive therapies (azathioprine and mycophenolic acid) and monoclonal antibodies (rituximab and tocilizumab); Wilcoxon matched pairs signed rank test. Data are presented as median and IQR. Each dot represents a single individual. ARR = annualized relapse rate; IQR = interquartile range; IST = immunosuppressive therapies; mAb = monoclonal antibodies; MOG+ = CRION patients positive for antimyelin oligodendrocyte glycoprotein antibody; MOG− = CRION patients negative for antimyelin oligodendrocyte glycoprotein antibody.

By contrast, immunosuppressive therapy in the MOG− CRION subgroup was associated with a reduction in ARR (n *=* 12; median [IQR] before vs after 1 [0–2] vs 0 [0–1], *p =* 0.063). Monoclonal antibody therapy was used less frequently (n *=* 5) but showed a comparable trend toward ARR reduction (median [IQR] 1 [0.5–2] before vs after 0 [0-0], *p =* 0.125; Figure 4B).

Subgroup analysis revealed that the positive response to monoclonal antibodies in MOG+ CRION was driven by the effect of tocilizumab (n *=* 11; median [IQR] before: 2 [1–3] vs after: 0 [0–1], *p =* 0.023; eFigure 5C). By contrast, neither rituximab (n *=* 10) nor azathioprine (n *=* 8) or mycophenolic acid (n *=* 3) was associated with a reduction of ARR in the MOG+ CRION subgroup (median [IQR] before vs after rituximab: 1 [1–3.3] vs 1.5 [0–3], *p =* 0.617; before vs after azathioprine: 2 [0.3–3.8] vs 2 [0.3–3], *p* > 0.999; before vs after mycophenolic acid: 2 [0–2] vs 1 [0–3], *p* > 0.999; eFigure 5, A–B, and D). In the MOG− subgroup, individuals receiving either rituximab (n *=* 4) or azathioprine (n *=* 9) demonstrated a favorable trend (median [IQR] ARR before vs after rituximab: 1 [0.3–1.8] vs 0 [0-0], *p =* 0.250; before vs after azathioprine: 1 [0–1.5] vs 0 [0–1], *p =* 0.250; eFigure 5, A–B). The limited use of mycophenolic acid (n *=* 2) and tocilizumab (n *=* 1) precluded further analysis. Treatment selection in the patient receiving IL-6 blocking therapy was guided by evidence of elevated CSF IL-6 levels at disease onset (11.4 pg/µL; the 95th percentile in controls is 7.0 pg/mL). To further illustrate treatment response over time, a 12-month survival analysis was performed, demonstrating the relapse-free probability under the respective therapies (eFigure 6).

## Discussion

Owing to its rarity and the lack of systematic clinical data, CRION remains an underexplored condition, posing substantial diagnostic and therapeutic challenges. In this multicenter retrospective longitudinal study, we compared patients with MOG-IgG-seropositive with MOG-IgG-seronegative CRION and found differences both in disease characteristics and treatment responses between the 2 groups.

MOG+ CRION patients demonstrated higher disease activity, characterized by elevated relapse rates 12 months before treatment initiation and a tendency toward shorter intervals between relapses compared with the MOG− subgroup. Notably, the overall ARR in the CRION cohort appeared higher compared with the ARR range previously reported for MOGAD in general.^14^ In addition, papilledema was more frequently observed in MOG+ CRION patients than in the MOG− group. This may reflect a more pronounced anterior optic nerve involvement in MOG+ CRION, consistent with an increased susceptibility to optic disc edema in MOGAD.

Recurrent episodes of ON lead to cumulative damage to the optic nerve, constituting a key risk factor for poor VA outcomes.^16-18^ Despite prompt treatment and favorable steroid responsiveness, visual acuity declined with increasing numbers of ON episodes across subgroups, reinforcing the importance of relapse prevention as a central therapeutic goal in the management of CRION. In addition, OCT revealed significant retinal thinning, with progressive pRNFL and GCIPL atrophy in both MOG+ and MOG− CRION patients over time, with no significant intergroup differences. These retinal changes are most likely attributable to sustained relapse activity (median 0.8 ON attacks/year) during the follow-up period, which prevented meaningful assessment of relapse-independent structural progression.

Although some individuals suffered severe and irreversible vision loss, overall VA outcomes were more favorable than previously reported.^2^ This disparity may reflect diagnostic advances enabling earlier identification of CRION, as well as autoantibody-based reclassification, excluding AQP4-IgG-positive NMOSD cases typically associated with worse VA outcomes. For example, one study reported an increase in AQP4-IgG seropositivity from 5% to 22% following reanalysis of serum samples from a previously characterized CRION cohort.^7^ The introduction of steroid-tapering protocols in atypical ON and the increasing use of monoclonal antibodies, particularly in MOG+ CRION individuals, may further contribute to better long-term outcomes. Finally, our analysis included only assessments performed >8 weeks after symptom onset. Differences in follow-up duration, clinical evaluation, and data reporting across previous studies may account for the observed discrepancies.

Further distinguishing features of MOG+ CRION cases included older age at onset, more extensive optic neuritis involvement, and a trend toward more frequent perineural involvement. Laboratory analysis revealed that markers of systemic autoimmunity and inflammation were more frequently detected in the MOG+ CRION subgroup compared with MOG− CRION patients. This included a higher frequency of antinuclear antibody positivity (47.6%), exceeding that observed in MOG− patients or previously reported values in MOGAD cohorts with mixed phenotypes (7–24%).^19-23^ In CSF, neutrophils were observed exclusively in MOG+ individuals and CSF IL-6 levels were significantly elevated compared with MOG− patients. These findings collectively suggest increased disease activity and IL-6 pathway involvement in the MOG+ CRION phenotype, consistent with previous reports on MOGAD,^19,20,24,25^ and may guide treatment strategies for this subgroup.

In clinical practice, treatment strategies for MOGAD, seropositive and seronegative NMOSD, and related disorders commonly include both immunosuppressive agents and monoclonal antibodies.^26-30^ Notably, in the MOG+ CRION subgroup, the use of monoclonal antibodies was associated with a significant reduction in ARR, in contrast to the limited response observed with conventional immunosuppressants. Subgroup analysis indicated that the positive effect was mainly driven by tocilizumab, aligning with previous reports for different MOGAD phenotypes.^31,32^ These findings require validation in larger, prospective studies. Neither rituximab nor azathioprine significantly reduced the ARR in MOG+ CRION, supporting emerging evidence that rituximab may be less effective in MOGAD.^33,34^ Given the retrospective design and limited sample size, these findings should be interpreted with caution and require confirmation in prospective studies.

In the MOG-IgG-negative CRION subgroup, both conventional immunosuppressive therapies and monoclonal antibodies were associated with a trend toward reduced relapse frequency, consistent with the limited evidence available from previous studies.^10,11^ Subgroup analysis suggested that azathioprine and rituximab may represent potential therapeutic options; however, these observations are based on small patient numbers and should therefore be considered exploratory. The restricted use of tocilizumab in MOG− CRION patients precluded assessment of effectiveness.

The initial description of CRION,^1^ along with subsequent studies, proposed that CRION is a distinct disease entity.^2,35^ However, after the discovery of MOG-IgG, others have suggested that CRION may represent a manifestation of MOGAD spectrum, based on high MOG-IgG seropositivity rates in 2 independent cohorts.^5,6^ In our longitudinal cohort, only 45% of patients were ultimately found to be MOG+ and were therefore reclassified MOGAD. This is a lower percentage compared with some previously published data,^5^ despite repetitive testing using cell-based assays. Although early relapse has been proposed as a clinical predictor of MOG-IgG seropositivity,^5^ approximately 27% of MOG− CRION patients also experienced early relapses. Overall, rather than supporting CRION as a single distinct immunopathologic entity, our findings support its use as a descriptive clinical syndrome. Within this framework, MOG-IgG-positive cases are likely best classified within the MOGAD spectrum, whereas MOG-IgG-negative cases probably represent a heterogeneous group with diverse underlying immunopathogenic mechanisms.

It is important that the absence of detectable antibodies, such as MOG-IgG, does not exclude autoimmune pathogenesis. Experimental models have demonstrated that MOG-specific T cells can induce optic neuritis and that spontaneous optic neuritis and myelitis may develop in the presence of MOG-specific T and B cells even in the absence of secreted anti-MOG antibodies.^36^ Furthermore, experimental autoimmune encephalomyelitis induced by MOG peptide is driven by MOG-specific T cells in the absence of B cells and antibodies, highlighting the potential central role of cellular immunity in MOG-directed autoimmunity.^37^ These observations raise the possibility that cellular autoimmune mechanisms, including MOG-specific T-cell responses, may contribute to the pathogenesis of at least a subset of patients with MOG-IgG-negative CRION. Further molecular and immunologic characterization of seronegative CRION phenotypes will be essential to define these mechanisms, refine disease classification, and guide individualized treatment strategies.

This study's retrospective, multicenter design has several limitations, most notably potential selection, and referral bias from tertiary center recruitment. The lack of standardized diagnostic timing, treatment protocols, and monitoring, specifically variable steroid-tapering, may have influenced relapse intervals and visual outcomes. Furthermore, missing data (e.g., MRI and CD20 depletion) and small treatment subgroups reduce statistical power and limit conclusions on comparative effectiveness. Diagnostic gaps, including the absence of CSF-restricted MOG-IgG testing, tissue biopsies, routine screening for GFAP-Ab, or genetic testing, may have resulted in the misclassification of rare cases (e.g., hereditary optic neuropathies). Consequently, these findings should be interpreted as exploratory and hypothesis-generating.

Despite these limitations, this study offers valuable insights into clinical profiles and treatment responses of MOG+ compared with MOG− CRION patients, suggesting that these subgroups may benefit from distinct treatment approaches. Further research in larger cohorts on the clinical, paraclinical, immunologic, and therapeutic response profiles of CRION subgroups is essential to guide personalized treatment strategies and prevent vision loss.

## Glossary

ANA: antinuclear antibody
AQP4: aquaporin-4
ARR: annualized relapse rate
CRION: chronic relapsing inflammatory optic neuropathy
GCIPL: ganglion cell layer and the inner plexiform layer
IQR: interquartile range
IVIG: IV immunoglobulins
MOG−: MOG-IgG seronegative
MOG+: MOG-IgG positivity
MOGAD: MOG antibody-associated disease
MOG-IgG: myelin oligodendrocyte glycoprotein antibodies
NLR: neutrophil-to-lymphocyte ratio
OCT: optical coherence tomography
ON: optic neuritis
pRNFL: peripapillary retinal nerve fiber layer
VA: visual acuity
